# Supplementary material for: Estimating infection prevalence using the positive predictive value of self-administered rapid antigen diagnostic tests: An exploration of SARS-CoV-2 surveillance data in the Netherlands from May 2021 to April 2022
Source: PLoS One. 2024 Feb 13;19(2):e0298218. doi: 10.1371/journal.pone.0298218 (PMC10863887; doi:10.1371/journal.pone.0298218)
Supplement: S1 File — (PDF) [file pone.0298218.s001.pdf]

S1 File. Formula used in our sensitivity analysis

- Total population = Pop
- Prevalence = prev
- True prevalence = True Prevalence()
- Estimated prevalence = Estimated prevalence()
- Individual with COVID-19 like symptoms and SARS-CoV-2 positive = Symp+ SARS-CoV-2+
- Individual without COVID-19 like symptoms and SARS-CoV-2 positive = Symp- SARS-CoV-2+
- Individual with COVID-19 like symptoms and SARS-CoV-2 negative = Symp+ SARS-CoV-2-
- Individual without COVID-19 like symptoms and SARS-CoV-2 negative = Symp- SARS-CoV-2-
- Proportion of individuals infected with SARS-CoV-2 that develop symptoms = Prop Symp+ SARS-CoV-2+
- Proportion of population with COVID-like symptoms and SARS-CoV-2 negative = Prop Symp+ SARS-CoV-2-
- Sensitivity of an Ag-RDT in symptomatic individuals = Sens symp+
- Sensitivity of an Ag-RDT in asymptomatic individuals = Sens symp-
- Specificity of an Ag-RDT = Spec
- Test propensity = p()
- Number of individuals in the population= n()
- Number of individuals that gets tested = ntested()
- Positive predictive value = PPV()

#Totals in population

n(Symp+ SARS-CoV-2+) = Pop * Prev * Prop Symp+ SARS-CoV-2+

n(Symp- SARS-CoV-2+) = Pop * Prev * (1-Prop Symp+ SARS-CoV-2+)

n(Symp+ SARS-CoV-2-) = Pop – (Pop*Prev) * Prop Symp+ SARS-CoV-2-

n(Symp- SARS-CoV-2-) = Pop – (Pop*Prev) * (1-Prop Symp+ SARS-CoV-2-)

#Numbers that get tested

ntested(Symp+ SARS-CoV-2+) = n(Symp+ SARS-CoV-2+) * p(Symp+ SARS-CoV-2+)

ntested(Symp- SARS-CoV-2+) = n(Symp- SARS-CoV-2+) * p(Symp- SARS-CoV-2+)

ntested(Symp+ SARS-CoV-2-) = n(Symp+ SARS-CoV-2-) * p(Symp+ SARS-CoV-2-)

ntested(Symp- SARS-CoV-2-) = n(Symp- SARS-CoV-2-) * p(Symp- SARS-CoV-2-)

#Positive predictive value and estimated prevalence

PPV(symp+) = (ntested(symp+ SARS-CoV-2+) * Sens symp+) / (ntested(symp+ SARS-CoV-2+) * Sens symp+) + (ntested(symp+ SARS-CoV-2- * (1- Specificity))

PPV(symp-) = (ntested(symp- SARS-CoV-2- * Sens symp-) / (ntested(symp- SARS-CoV-2- * Sens symp-) + ntested(symp SARS-CoV-2+ * (1-Specificity))

Estimated prevalence(symp+) = (1 - Specificity) / ((Sens symp+ / PPV(symp+) – Sens symp+ – Specificity + 1)

Estimated prevalence(symp-) = (1 - Specificity) / ((Sens symp- / PPV(symp-) – Sens symp- – Specificity + 1)

#True prevalence

True prevalence(symp+) = n(Symp+ SARS-CoV-2+) / (n(Symp+ SARS-CoV-2+) + n(Symp+ SARS-CoV-2-)

True prevalence(symp-) = n(Symp- SARS-CoV-2+) / (n(Symp- SARS-CoV-2+) + n(Symp- SARS-CoV-2-)
