# Supplementary material for: Estimating infection prevalence using the positive predictive value of self-administered rapid antigen diagnostic tests: An exploration of SARS-CoV-2 surveillance data in the Netherlands from May 2021 to April 2022
Source: PLoS One. 2024 Feb 13;19(2):e0298218. doi: 10.1371/journal.pone.0298218 (PMC10863887; doi:10.1371/journal.pone.0298218)
Supplement: S1 Table — (PDF) [file pone.0298218.s003.pdf]

S1 Table. Correlation between COVID-related hospital admissions and estimated SARS-CoV-2 prevalence in the population at different points in time

Correlation (p value)	Correlation Hospital admissions and current prevalence	Correlation Hospital admissions and prevalence one week earlier	Correlation Hospital admissions and prevalence two weeks earlier	Correlation Hospital admissions and prevalence three weeks earlier
Sympomatic individuals	0,52*	0,58*	0,6*	0,57*
Asymptomatic individuals	0,58*	0,66*	0.69*	0,67*
Symptomatic individuals Omicron period (3 January 2022 and onwards)	0,72 (p=0,003)	0,84*	0,66 (p=0,01)	0,46 (p=0.09)
Asymptomatic individuals Omicron period (3 January 2022 and onwards)	0,72 (p=0,04)	0,74 (p=0,002)	0,63 (p=0,02)	0,51 (p=0,06)

* p <0,001
