# Supplementary figures and images for: Estimating infection prevalence using the positive predictive value of self-administered rapid antigen diagnostic tests: An exploration of SARS-CoV-2 surveillance data in the Netherlands from May 2021 to April 2022
Source: PLoS One. 2024 Feb 13;19(2):e0298218. doi: 10.1371/journal.pone.0298218 (PMC10863887; doi:10.1371/journal.pone.0298218)

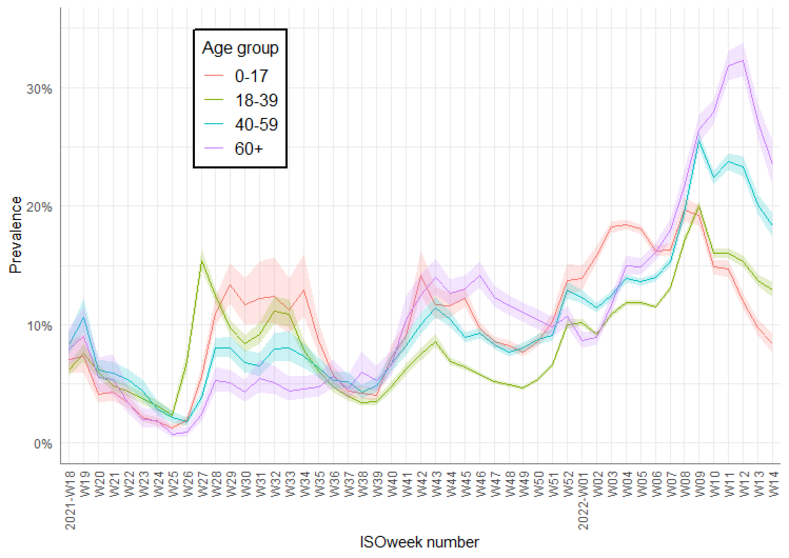

Supplement: S1 Fig — (TIF) [file pone.0298218.s004.tif]

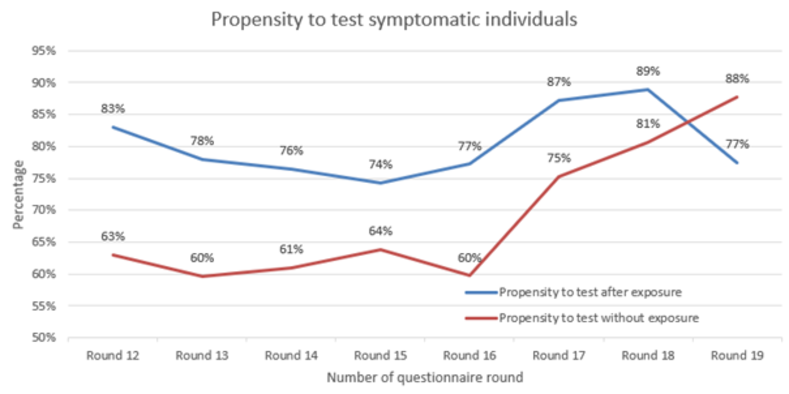

Supplement: S2 Fig — (TIF) [file pone.0298218.s005.tif]

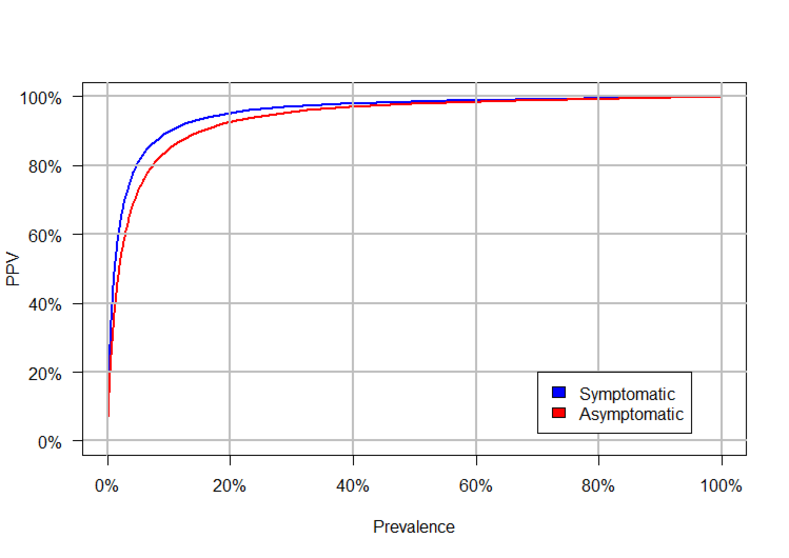

Supplement: S3 Fig — Sensitivity was set at 0.8 for symptomatic individuals, 0.5 for asymptomatic individuals and specificity at 0.99 for both groups. (TIF) [file pone.0298218.s006.tif]
