# Supplementary material for: Estimating infection prevalence using the positive predictive value of self-administered rapid antigen diagnostic tests: An exploration of SARS-CoV-2 surveillance data in the Netherlands from May 2021 to April 2022
Source: PLoS One. 2024 Feb 13;19(2):e0298218. doi: 10.1371/journal.pone.0298218 (PMC10863887; doi:10.1371/journal.pone.0298218)
Supplement: Attachment — Submitted filename: Response to editors PLOS ONE.docx [file pone.0298218.s007.docx]

We would like to thank the editors for their review and their comments. Below you will find a list of the questions posed with our answer. We updated our main body of text if necessary.

When submitting your revision, we need you to address these additional requirements.

*1. Please ensure that your manuscript meets PLOS ONE's style requirements, including those for file naming. The PLOS ONE style templates can be found at 
https://journals.plos.org/plosone/s/file?id=wjVg/PLOSOne_formatting_sample_main_body.pdf and 
https://journals.plos.org/plosone/s/file?id=ba62/PLOSOne_formatting_sample_title_authors_affiliations.pdf*

We made these changes

*2. In your Data Availability statement, you have not specified where the minimal data set underlying the results described in your manuscript can be found. PLOS defines a study's minimal data set as the underlying data used to reach the conclusions drawn in the manuscript and any additional data required to replicate the reported study findings in their entirety. All PLOS journals require that the minimal data set be made fully available. For more information about our data policy, please see http://journals.plos.org/plosone/s/data-availability.*

*Upon re-submitting your revised manuscript, please upload your study’s minimal underlying data set as either Supporting Information files or to a stable, public repository and include the relevant URLs, DOIs, or accession numbers within your revised cover letter. For a list of acceptable repositories, please see http://journals.plos.org/plosone/s/data-availability#loc-recommended-repositories. Any potentially identifying patient information must be fully anonymized.*

*Important: If there are ethical or legal restrictions to sharing your data publicly, please explain these restrictions in detail. Please see our guidelines for more information on what we consider unacceptable restrictions to publicly sharing data: http://journals.plos.org/plosone/s/data-availability#loc-unacceptable-data-access-restrictions. Note that it is not acceptable for the authors to be the sole named individuals responsible for ensuring data access.*

*We will update your Data Availability statement to reflect the information you provide in your cover letter.*

We provided a minimal dataset in the supplementary materials, with information for each specific week on symptom status and age. All analyses and visual representations in our study were derived from this fully anonymized dataset.

*3. Please include your full ethics statement in the ‘Methods’ section of your manuscript file. In your statement, please include the full name of the IRB or ethics committee who approved or waived your study, as well as whether or not you obtained informed written or verbal consent. If consent was waived for your study, please include this information in your statement as well.*

We added the ethical statement in the method section

4. Please include captions for your Supporting Information files at the end of your manuscript, and update any in-text citations to match accordingly. Please see our Supporting Information guidelines for more information: http://journals.plos.org/plosone/s/supporting-information.

We added these captions

*Results
(lines 154-61): I find it quite interesting that the correlation is positively higher for asymptomatic cases than for symptomatic cases. Could the authors explain this finding?*

We acknowledge this observation, and although we cannot offer a definitive explanation, we can put forth a plausible hypothesis. During the period from 2021-W33 to 2021-W40, we noted a significant decrease in the Positive Predictive Value (PPV) – and consequently, the estimated prevalence – among symptomatic individuals. Conversely, asymptomatic infections and hospitalizations experienced only a marginal decline. Moreover, a drop in PPV and estimated prevalence coincided with a sharp increase in hospital admissions from 2021-W42 to 2021-W49. We believe that a seasonal upswing in COVID-like symptoms caused by other pathogens may have contributed to this pattern. Additionally, during this period, the guidance to use self-tests exclusively when asymptomatic may have prompted individuals with multiple or severe symptoms to directly seek assistance from public health services, bypassing the use of Ag-RDTs. On the other hand, those with less distinctive symptoms (and consequently, a lower likelihood of SARS-CoV-2 infection) may have opted for self-testing instead. These factors are less relevant for asymptomatic individuals, potentially accounting for the stronger correlation observed in that group.

In the omicron period, we speculate that individuals with pronounced symptoms who received a positive Ag-RDT result may have refrained from seeking confirmation at public health services, further attenuating the correlation compared to asymptomatic individuals.

We added one sentence in our discussion to mention this fact.

*Under line (174-5) “An underestimation of the sensitivity and specificity of the Ag-RDT’s leads to an overestimation of the prevalence” : Is this sentence reporting the table results or is this a discussion point? If the former is true, I’m not sure if this is reporting the last 2 rows of Table 2? Also, I would suggest adding the sensitivity & specificity values for the baseline as a Table footnote.*

This sentence refers to the table results. In the last two rows the table shows that increasing the true sensitivity and true leads to an overestimation of the prevalence using our method of analysis. We agree a footnote clarifies our statement and we added this footnote.

*Discussion
While the relationship between PPV and severity is emphasized in the introduction (and which is likely the basis why COVID-related hospital admissions data were collected), I feel there is a lack in discussing this point in the discussion section. One reason could be the structure of the section. This is especially true for the 6th paragraph, I’m quite confused why hospital admissions are being compared with prevalence. I would suggest to consider revising the discussion section to better clarify the points intended by the authors.*

We agree with your statement. The central query we aimed to address in our paper is whether the Positive Predictive Value (PPV) of self-tests holds the potential to serve as a tool for estimating the underlying prevalence and so to contribute to discussions on disease severity. Our findings led us to conclude that while our current estimate proves valuable in discerning fundamental shifts in prevalence, it (currently) lacks the necessary precision for robust estimations of this prevalence to be used in context of hospitalization rates and infer disease severity. We concur that hospital admissions, being an incidence rate, are not a direct indicator of prevalence. Various other elements, such as age, immunity resulting from vaccination or prior infections, and evolving virulence across different variants, also exert influence on the number of hospital admissions. However we still think that hospital admissions is the best comparator available linked to the prevalence of infections and were only used to examine a correlation between our estimates of the prevalence based on PPV as an external validation.

We changed our introduction and part of our discussion to better reflect the goals of our study and improved the overall structure.

*Suggest to include a brief explanation, or a timeline figure, on the public health intervention measures implemented in the Netherlands during the study period. As this would help readers understand better the local context and also the points raised in the discussion section.*

We agree and we added a brief overview of measures in the Netherlands in the method section.

*Minor comments:
- Abstract (2nd paragraph, 2nd sentence): Please clarify it’s the overall positivity rate of..?*

We changed this to overall PPV.

*- The manuscript has some minor punctuation mistakes; I suggest to proofread carefully*

We fixed some punctuation mistakes.

*- Table 1 caption: I don’t understand this, suggest to clarify: “***= All individuals with at least one vaccination that are not covered by the fully vaccinated category.”*

These are individuals that only received one vaccination or received a second vaccination but were still within a 14-28 day window so they were not considered fully vaccinated. We changed the text in the table to clarify what we mean.

*- Table 2 (columns 5 & 6): Please check the headings if this should this be correctly read as “Propensity to test SARS-CoV-2 + / SARS-CoV-2 - ?” I would suggest to use positive & negative to avoid confusion, or add a footnote to clarify.*

We agree and changed this in the text

*Reviewer #1: Generally, well written manuscript. with the vailability of vaccination data for more than two thirds of study participants it would be great to get a sense of how vaccination coverage over time impacted estimated prevalence/ PPV in the vaccinated compared to the unvaccinated groups*

We also found this an interesting question and in fact, we delved into this matter. During our research we concluded that without data on test propensity interpreting results of PPV in the context of prevalence is challenging, as a difference in PPV could occur due to differences in testing between vaccinated and unvaccinated individuals or due to a true difference in prevalence. Furthermore a difference in prevalence should depend on to which extent the vaccinated and unvaccinated socially mix as these individuals might cluster together and we don’t have data for this analysis. As we conclude in our paper PPV in the context of prevalence can be used to gauge underlying trends but is not yet suitable for precise interpretations of a subgroup analysis such as you suggest on vaccinated versus unvaccinated individuals as this necessitates additional data as mentioned above.

For your interest we will give a rough overview of our analysis and results on vaccination status.

Interestingly, the overall Positive Predictive Value and estimated prevalence (PPV) differed between vaccinated and unvaccinated individuals. From the period 2021-W27 (the first week data was available on vaccination status for vaccinated individuals) , unvaccinated individuals displayed a slightly higher PPV and estimated prevalence, but this trend reversed (approximately in 2021-W44) when the delta variant was dominant and subsequently remained when omicron became dominant. Given that we did not have data on test propensity we could not make a valid interpretation of this difference and why the difference reversed.
